# Gradient Confinement Induced Uniform Tensile Ductility in Metallic Glass

**DOI:** 10.1038/srep03319

**Published:** 2013-11-25

**Authors:** X. L. Lu, Q. H. Lu, Y. Li, L. Lu

**Affiliations:** 1 Shenyang National Laboratory for Materials Science, Institute of Metal Research, Chinese Academy of Sciences, 72 Wenhua Road, Shenyang, 110016, P. R. China; 2 Department of Materials Science and Engineering, Faculty of Engineering, National University of Singapore, Singapore 117576

## Abstract

Metallic glass (MG) generally fails in a brittle manner under uniaxial tension loading at room temperature. The lack of plastic strain of MG is due to the severe plastic instability via the easily formed one dominate shear band. There have been several approaches to improve the ductility in MG, but achieving uniform tensile ductility for monolithic MG in bulk size remains a challenge. Here we demonstrate a uniform tensile ductility of 12% achieved in a micrometer scale Ni-P amorphous film coated on a Ni substrate with gradient structure. Instead of a single run-away shear band, such a gradient structure generates massive extensive multiple shear bands in the film, leading to a record high tensile ductility in MG. The present finding highlights a novel route for achieving uniform tensile ductility in monolithic metallic glass with bulk size.

The main plastic deformation mechanism in metallic glasses (MGs) is through shear localization into narrow band^1^,^2^,^3^. Due to their inability to suppress the formation of shear bands, such localization often results in runaway of one dominate shear band, leading to catastrophic failure and exhibiting macroscopically brittle behavior. Because of this, the unconstrained free standing monolithic BMG plate or film shows no or little tensile ductility^4^,^5^,^6^,^7^,^8^. The exceptional cases of uniform deformation, or no shear banding have been reported only in nano-sized pillars^9^ or thin films^10^,^11^ or in the necking region of the sample^12^. Other approaches have also been used to improve the room temperature tensile ductility in MGs, for example adding dispersive inclusions in the amorphous matrix^13^,^14^,^15^, and reducing sample size (such as multilayer nanolaminate)^16^,^17^,^18^,^19^,^20^, and increasing machine stiffness^21^,^22^. However, for the monolithic metallic glass, particularly in the bulk or at least in macro-sized specimens, tensile ductility remains a challenge.

Nevertheless, delocalizing strain can be achieved if the brittle materials are constrained by a plastic substrate^23^,^24^. For example, in nanocrystalline (NC) counterparts which usually rupture at a small strain under tension, confinement by a ductile substrate is one of effective ways to suppress the strain localization under tension tests. Lu *et al.*^25^ showed that NC Cu films well-bonded on to a ductile polymer substrate can be elongated beyond 50% tensile strain before failure. On the other hand, Fang *et al.*^26^ demonstrated that gradient nano-grained (GNG) surface Cu layer can sustain a uniform tensile strain of 30% without any localized deformation and cracks when it was confined by a coarse grained substrate. Above experiments had demonstrated that efficient confinement of plastic deformation by the substrate can suppress the strain localization and delay the catastrophic failure, leading to a considerable tensile elongation in the NC metals. This understanding enlightens the possibility to achieve tensile elongation in metallic glass as long as strain localization in the specimen can be constrained, for example, through the GNG substrate.

In this work, we choose to investigate the tension behaviors of Ni-P amorphous film with a gradient substrate confinement. A spatial gradient nano-grained structure in nickel rod substrate was prepared by means of surface mechanical grinding treatment (SMGT) material. And then a Ni-P amorphous film was coated on the top surface. Subsequent tensile tests revealed a 12% uniform tensile ductility on macro-sized amorphous films achieved through the generation of high density of shear bands in a tensile specimen has not been reported before.

## Results

**Figure 1:**
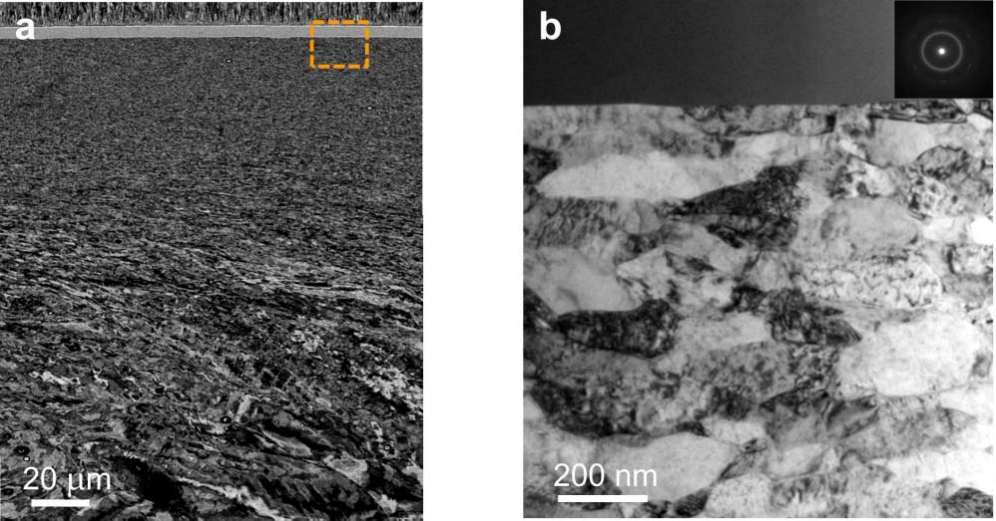
Transverse-sectional microstructure of GNG Ni substrate with Ni-P amorphous film. (a), SEM image of a typical GNG Ni substrate with Ni-P amorphous film. (b), A bright-field TEM image of microstructure near the interface, and the inset shows the electron diffraction of the Ni-P film.

Figure 1a shows the hierarchical structure where a 6 μm thick Ni-P amorphous film is coated on the GNG Ni substrate. Elongated grains with average transverse grain size of about 65 nm and longitudinal size of about 174 nm were found in the top 20 μm of the GNG Ni substrate, which increased to more than 300 nm in a depth of 20 to 100 μm. Beneath these, there is a layer of deformed structure with increasing grain size from 300 nm to the coarse grain core with grain size of tens micrometers, as shown in Fig. 1. Fig. 1b shows the TEM transverse sectional microstructure at the interface between the Ni-P amorphous layer and the GNG Ni substrate. The interface is distinguished sharp, straight and clean, showing that the film is well adhered to the substrate. The amorphicity of the Ni-P film is further confirmed by the TEM diffraction (the inset in Fig. 1b) and X-ray (Supplementary Information Fig. S2).

**Figure 2:**
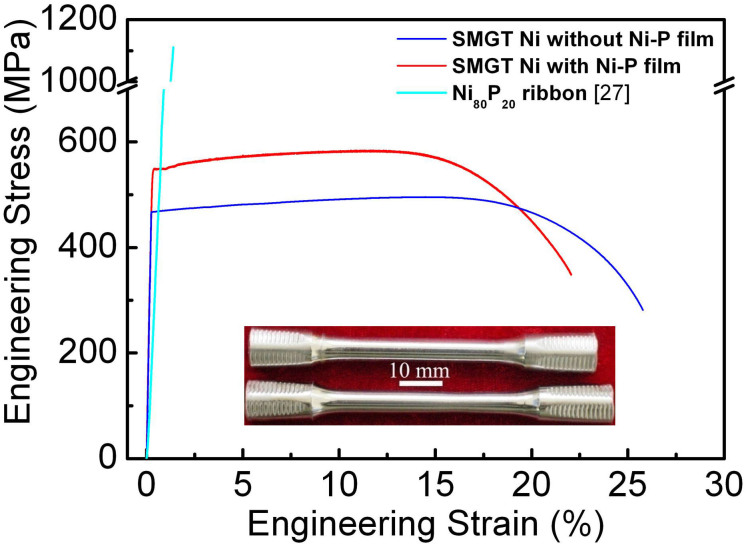
Quasi-static tensile stress versus strain curves. Typical Engineering tensile stress-strain curves of the Ni-P film with GNG Ni substrate. For comparison, the s-s curves of a GNG Ni sample without Ni-P coating and free-standing Ni_80_P_20_ are also included^27^.

The typical engineering tensile behaviors of the samples with and without Ni-P coating are shown in Fig. 2. Tensile curve of a free-standing Ni_80_P_20_ ribbon prepared by melt-spinning is also included in Fig. 2 for comparison^27^. Apparently, the ribbon shows high strength but with no plasticity. The sample with Ni-P film coating exhibited a much enhanced tensile strength (yield strength at 0.2% offset, *σ_y_*, of 550 MPa and ultimate tensile strength (*σ_uts_*) of about 580 MPa), compared with the GNG Ni sample (*σ*_y_ = 470 MPa, and *σ*_uts_ = 480 MPa, respectively). A considerable tensile uniform strain *ε_u_* as high as 12% was detected in the sample with Ni-P film. Such an impressive uniform tensile ductility has never been reported for MG materials in the literature. Interestingly, the uniform tensile strain of the samples with Ni-P coating is comparable to that of the GNG Ni substrate. A slight weak strain hardening, comparable to the GNG Ni substrate, appears in the major plastic deformation stage for the sample with Ni-P film. That indicates dislocation accumulation, which may solely come from the strain hardening of the GNG Ni substrate. The insets in Fig. 2 are the macroscopic images of the GNG Ni sample (top) and the sample with Ni-P film (bottom) with a tensile strain of 15%. The shining surfaces indicate that there are no clear differences in surface morphology between those two samples from macro-scale observations.

**Figure 3:**
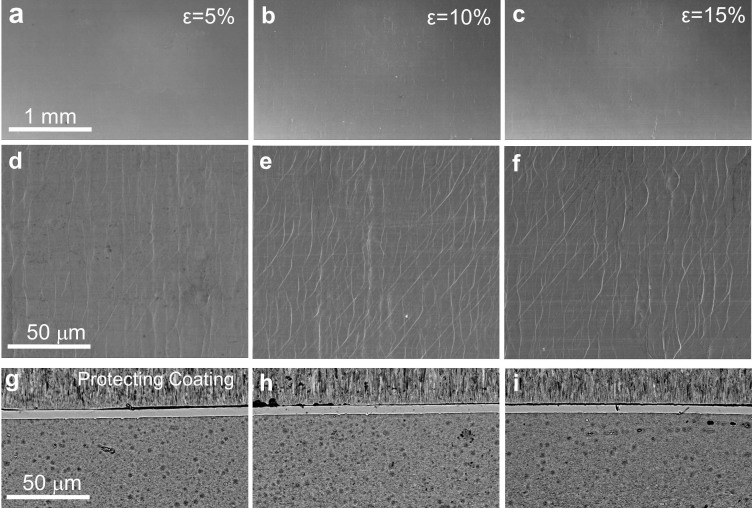
Surface and transverse-sectional images of Ni gradient structure with Ni-P amorphous film after tension. SEM images of the amorphous film surface with engineering strain of 5% (a, d), 10% (b, e), 15% (c, f). (d–f) is magnified images. (g–i), Transverse sectional SEM images with true strain of 4.8% (g), 9.6% (h), 14.1% (i) calculated by contraction of cross section area.

In order to investigate the deformation behavior of Ni-P film with GNG substrate, tensile tests with different elongations were designed. The samples were loaded to a constant strain, namely 5%, 10%, 12.5% and 15%, respectively. The engineering tensile stress-strain curves for the samples with Ni-P film are shown in SI Fig. S3. The well overlapped stress-strain curves of the samples with different strains suggested a good repeatability. Fig. 3 illustrates the surface morphologies of the samples subjected to 5 to 15% elongation. No surface cracking was observed in the sample with strain less than 10% shown at low magnification (Figs. 3a–c). Dense multiple shear bands were observed at high magnification (Figs. 3d–f). Few cracks can be seen in the Ni-P film with 15% strain from surface and transverse-sectional SEM images (Figs. 3c & 3i). It is noticed that such cracking may be due to the localized necking effect in the GNG Ni substrate beneath the film. If the substrate can be further strained without necking, it is foreseen that further tensile elongation of the Ni-P film beyond 12% without cracking is possible. The above microscopic structures further confirmed that the Ni-P films sustain a uniform tensile elongation associated with the formation of multiple shear bands.

**Figure 4:**
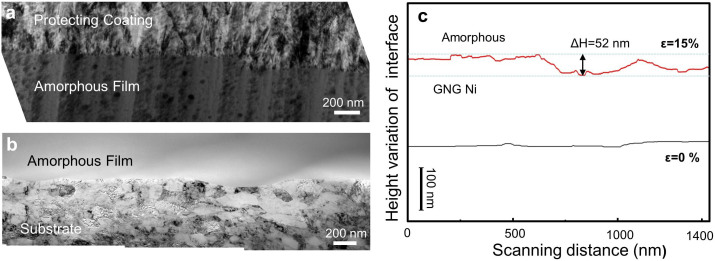
Transverse-sectional microstructure of GNG Ni substrate with Ni-P amorphous film after tension. TEM images of amorphous film top surface (a) and interface (b) between the Ni-P film and substrate with a true strain of 15%. (c), The height variations of the interface between the Ni-P film and GNG Ni substrate before (0%) and after tension tests (15%). An additional Ni film was deposited outside the Ni-P film to cover its free surface after tensile testing.

The transverse–sectional TEM observations indicated that the tensile-deformed Ni-P is intact as shown in Fig. 4a, while the typical shear steps (~200 nm) are clearly seen on the amorphous film free surface after ~15% tension. On the other hand, a smooth interface between the Ni-P film and GNG Ni substrate became obviously uneven with a fluctuation more than 50 nm in comparison with that before tension (less than few nanometer) (Fig. 4c). Yet the film is still adhered to the substrate firmly and no delamination was observed at the interface (Fig. 4b), indicative of tensile deformation occurred in the Ni-P film and GNG substrate cooperatively.

**Figure 5:**
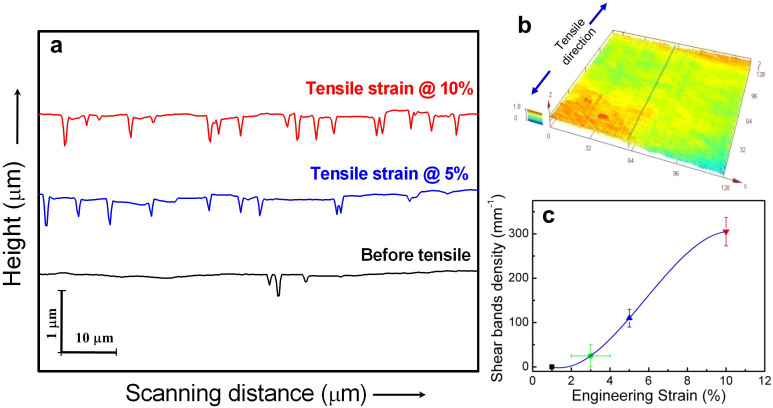
Multiple shear bands measured by confocal laser scanning microscope. (a) Measured surface height variation profiles of the tensile sample before and after tension with a strain of 5% and 10%, respectively. (b) A 3D image of observed surface of Ni-P amorphous film after tension. (c) Shear bands density increases with the increase of engineering strain.

The offset of shear bands in Ni-P films with different tensile strains were further measured using a confocal laser scanning microscope (CLSM) (Fig. 5). Comparing the glossy surface of Ni-P film before tensile, shear bands nucleated randomly throughout the film. The average magnitude of the offset (h) of each shear band is around 200 nm, which is well consisted with TEM observation (Fig. 4a). The spacing between two individual shear bands gets smaller and smaller with increasing tensile strain. The density of shear bands (D) increased from 110 mm^−1^ for the 5% sample to 305 mm^−1^ for the 10% sample, estimated from the corresponding decrease in the average spacing from 9.1 to 3.3 μm, respectively. The total shear strain (γ) due to shear banding can be simply estimated by γ = h × D, as 2.2% in 5% sample and 6.1% in 10% sample, respectively (Fig. 5c).

## Discussion

To explain the uniform tensile ductility of Ni-P amorphous film, we consider the key role played by the GNG Ni substrate with high plastic deformation and minimum surface roughness. In contrast to the brittle deformation of the free-standing NC materials, due to the confinement of GNG Ni structure, the strain localization and early rupture of Ni top layer are effectively suppressed. The gradient nano-grained substrate is intrinsically ductile and its plastic deformation is dominated by a novel deformation mechanism. The mechanical driven grain growth associated with grain boundary activities of nanosized grains in the top surface, rather than the dominated dislocation activity in the traditional coarse grained metals, ensures the uniform plastic deformation and extraordinary plasticity of NC layer near the interface^26^. And most importantly, this deformation mechanism of GNG substrate can also ensure the interface not as rough as coarse-grained substrate. Such microscopically extraordinary uniform plastic deformability without any fracture in the top layer of the substrate delocalized deformation in the coated amorphous film as the stress concentration in the film is correspondingly suppressed and the early failure of the amorphous Ni-P film is subsequently avoided.

The uniform plastic deformation of film can also be due to the minimum surface roughness. The surface roughness of the Ni-P film after 15% tensile strain is found to be less than 200 nm. This is consistent with the small roughness of 100 nm observed on the surface of the GNG Cu after 20% tensile strain^26^. A smooth interface with minimum roughness between the Ni-P film and GNG Ni substrate is also observed from TEM observations, as indicated in Fig. 4b. Clearly, small roughness of the GNG substrate will lead to smoother interface, which in turn would decrease the stress concentration and strain localization and avoid delamination. All of these facts above help to reduce the catastrophic failure of plastic deformation of Ni-P amorphous film. For comparison, a Ni-P amorphous film was also deposited on a coarse-grained Ni substrate, where numerous large cracks with a very rough surface are frequently observed after tensile tests.

Well matched moduli between the film and the substrate would be another reason as that the two sides of interface can deform cooperatively. According to the study of plastic deformation of metal film on the polymer substrate, the stiffer the substrate is, the larger the rupture strain would be^28^. Here in this study, the Young's modulus of Ni is ~200 GPa, and the hardness of GNG Ni surface layer is as high as 4.9 GPa as measured by means of nano-indentation. Both of them are comparable to those of Ni-P amorphous film (160 GPa and 5.6 GPa) respectively. Finally, the well-bonded interface between Ni-P amorphous film and GNG Ni substrate may also be a key point in our study. A well bonded interface can accommodate the plastic deformations of the both sides of the interfaces. Poorly bonded interface makes the film readily to form early cracks at relatively small macroscopic strains by facilitating the co-evolution of strain localization and delamination.

Interestingly, we noticed that there is a discrepancy between the uniform tensile strains from stress-strain curve and the estimation shear strain due to the contribution by the multiple shear bands from the laser measurement. This difference could simply mean that the total plasticity may not be only accounted for by the visible shear offsets from CLSM (Fig. 5a). The offset of the multiple shear bands from different directions and the tiny shear bands (which may not be resolved by the CLSM) may contribute to the plastic strain as well. On the other hand, it may also imply that the body of the film has undergone some kind of plastic deformation. This “homogeneous” plastic deformation has also been reported by Guo *et al.*^10^ and Deng *et al.*^11^ in nano-sizes samples. The possible reasons for this are under further investigation.

In summary, a tensile strain up to 12% was achieved in the micrometer-sized Ni-P amorphous film with a confinement of GNG Ni substrate. As discussed above, in order to inhibit the runaway of one dominant shear band in the Ni-P amorphous film, some requirements on the technical choice of the substrate for the confinement of plastic deformation are essential, such as the well bonded interface with minimum interface roughness, the matched strength and moduli and the extraordinary uniform plastic deformability of GNG Ni substrate in macro and micro. All of these factors have helped to suppress localized stress concentration and strain localization. The GNG Ni right below the Ni-P amorphous film will be a perfect substrate for its uniform deformation, which inhibits the runaway of one dominate shear band and helps to distribute the stress/strain concentration and activate relative uniform multiple mini-shear bands. The results highlight a novel route for achieving uniform tensile ductility in monolithic metallic glass with bulk sample size.

## Methods

Commercial coarse grained Ni with purity of 99.7% was cut into dog-bone-shaped bar with a gauge diameter of 6 mm and length of 30 mm (SI Fig. S1) and then subsequently annealed at 700°C for 6 h to achieve a fully recrystallization microstructure. The specimens were processed by means of SMGT at room temperature to form a GNG structure (about tens nm at the top surface to tens micrometer in the core substrate in the gauge section). The top surfaces of GNG Ni substrate were slightly mechanically polished and activated by electrolysis. Then an amorphous Ni-P film was electrolessly deposited on the surface of the GNG Ni rod substrate at 85°C for 30 minutes (SI Fig. S2). The tensile tests were carried out on an Instron 8801 Testing System (MTS) at ambient temperature and with a strain rate of 2.5 × 10^−5^/s. A clip-on extensometer was used to calibrate and measure the strain of the samples upon tensile. The sample surfaces were examined by scanning electron microscope (SEM) and confocal laser scanning microscope (CLSM) before and after tensile tests, respectively. The microstructure of Ni-P amorphous and GNG Ni substrate were characterized by transmission electron microscope (TEM) from transverse sectional observations along the loading direction.

## Author Contributions

L.L. and Y.L. proposed the idea and designed the research plan. X.L.L. carried out the experiments and collected the data. L.L., Y.L. and X.L.L. analysis the data and wrote the paper. Q.H.L. and X.L.L. performed TEM investigation.

## Supplementary Material

Supplementary Information supplementary information
