# Supplementary material for: Gradient Confinement Induced Uniform Tensile Ductility in Metallic Glass
Source: Sci Rep. 2013 Nov 25;3:3319. doi: 10.1038/srep03319 (PMC3839029; doi:10.1038/srep03319)
Supplement: Supplementary Information [file srep03319-s1.pdf]

SUPPLEMENTARY INFORMATION

Title:

Gradient Confinement Induced Uniform Tensile Ductility in Metallic Glass

X.L. Lu [1], Q.H. Lu [1], Y. Li* [1,2] and L. Lu* [1]

[1]Shenyang National Laboratory for Materials Science, Institute of Metal Research, Chinese Academy of Sciences, 72 Wenhua Road, Shenyang, 110016, P.R. China

[2]Department of Materials Science and Engineering, Faculty of Engineering, National University of Singapore, Singapore 117576

Correspondence and requests for materials should be addressed to:
L.L (llu@imr.ac.cn); Y. L (liyi@imr.ac.cn)

1. Materials and gradient nano-grained (GNG) sample preparation

Surface mechanical grinding treatment (SMGT)[1] was used to synthesize a gradient nano-grained (GNG) surface layer on a coarse grain (CG)Ni substrate. Commercial CG Ni with purity of 99.7% was cut into dog-bone-shaped tensile bar with a gauge diameter of 6mm and length of 30mm of which the geometry is indicated in Fig. S1, and then subsequently annealed at 700°C for 6h for a fully recrystallizion microstructure. The average grain size of the as-annealed Ni is about19 μm. Then the tensile bars were processed by means of SMGT at room temperature. The SMGT processing parameters are as follows: rotating velocity of the sample v1=300 rpm, sliding velocity of the tool tip v2 = 30 mm min^{-1}, the preset penetration depth of the tooltip into the sample ap = 30 μm. A hemi-spherical WC/Co tool tip (with a radius of r = 5 mm) was used. For each sample, the SMGT process was repeated twelve times with the same processing parameters for achieving a thick and uniform GNG layer. Plastic deformation is rather uniform in the surface layer and the surface is shining and smooth with a small surface roughness (Ra ≈ 0.15 μm). No crack was identified in the surface of the SMGT processed samples.

2. Ni-P film deposition

Electroless deposition of the Ni-P amorphous coating on the GNG Ni substrates was processed by immersing the tension bar samples in a hypophosphate bath. The detail of the chemical composition is as follows: 20 g/l nickel sulfate, 30 g/l sodium hypophosphite, 30 g/l sodium acetate，12.5 ml/l lactic acid, and 1 mg/l lead acetate. The solution was maintained at a pH value of 4.5~5.0, a temperature of 85±2°C and a deposition time of 30 min. The thickness of the Ni-P film is about 6 μm measured from the cross-sectional SEM image. The XRD result shows a typical “broad peak” indicates the amophicity of Ni-P film (as indicated in Figure S2a, which is consistent with the previous studies that the structure is in amorphous state with high phosphorous content (8-14 wt. %)[2-4]. The chemical content of the Ni-P amorphous is: P is 9.57 wt. % and Ni is 90.43wt.%, as indicated by the EDX analysis in Figure S2b.

3. Tensile tests

Uniaxial quasi-static tensile tests were performed in an Instron 8801Testing System (MTS) at ambient temperature and a strain rate of $2.5x10^{-5}s^{-1}$. A clip-on extensometer was used to calibrate and measure the strain of the samples upon tensile.

4. XRD, TEM, SEM,EDX, CLSM experiments

X-ray diffraction (XRD) measurements were carried out on the sample surface using a Rigaku DMAX/2400 X-ray diffractometer with Cu Ka radiation. Transverse sectional Ni-P amorphous and Ni substrate interface were characterized by transmission electron microscope (TEM) images using an FEI Tecnai G^2 20 at an accelerating voltage of 200 KV. The top surfaces of samples were examined in a FEI Nova NanoSEM 430 field emission gun scanning electron microscope (SEM) with secondary electron imaging and the cross-sectional characterization was carried out with electron-channeling contrast (ECC) imaging using a VCD detector. Energy Dispersive X-ray analysis (EDX) were carried out on Oxford INCA X-act fixed on SEM 430.The shear bands on the surface after tensile tests were detected by using an Olympus 4000 confocal laser scanning microscope (CLSM). The height resolution in Z axe is 10 nm.

5. Supplementary Figures S1-S5

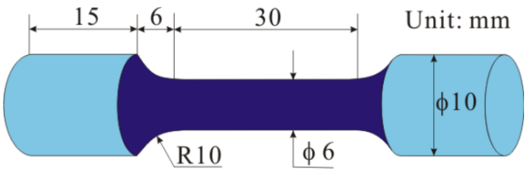

Figure S1. Illustration of the tensile bar sample geometry. The blue area indicated the GNG region deformed by SMGT process. The gauge dimension is Φ6 ×30 mm.

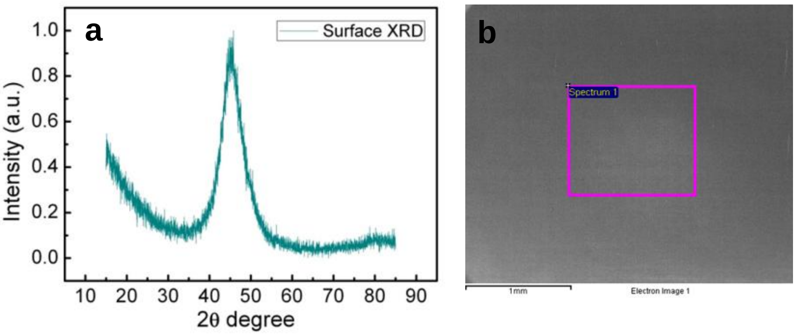

Element	Weight%	Atomic%
P K	9.57	16.70
Ni K	90.43	83.30

Figure S2. XRD and EDX analysis of the as-deposited Ni-P film

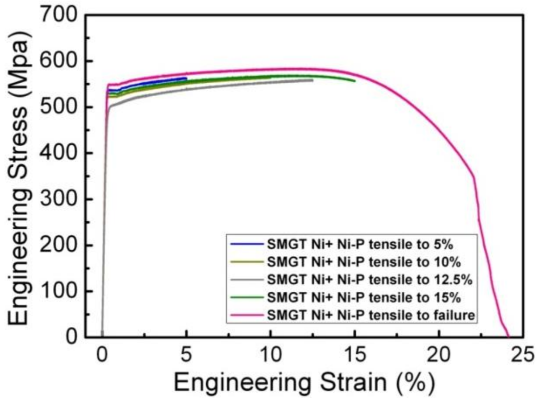

Figure S3. Engineering tensile stress-strain curves of GNG Ni with Ni-P film with different strains (5%, 10%, 12.5% and 15%, as indicated in the Figures),respectively. The slight differences among the s-s curves come from the depth variation of top GNG layer in SMGT processing and the gauge size measurement error.

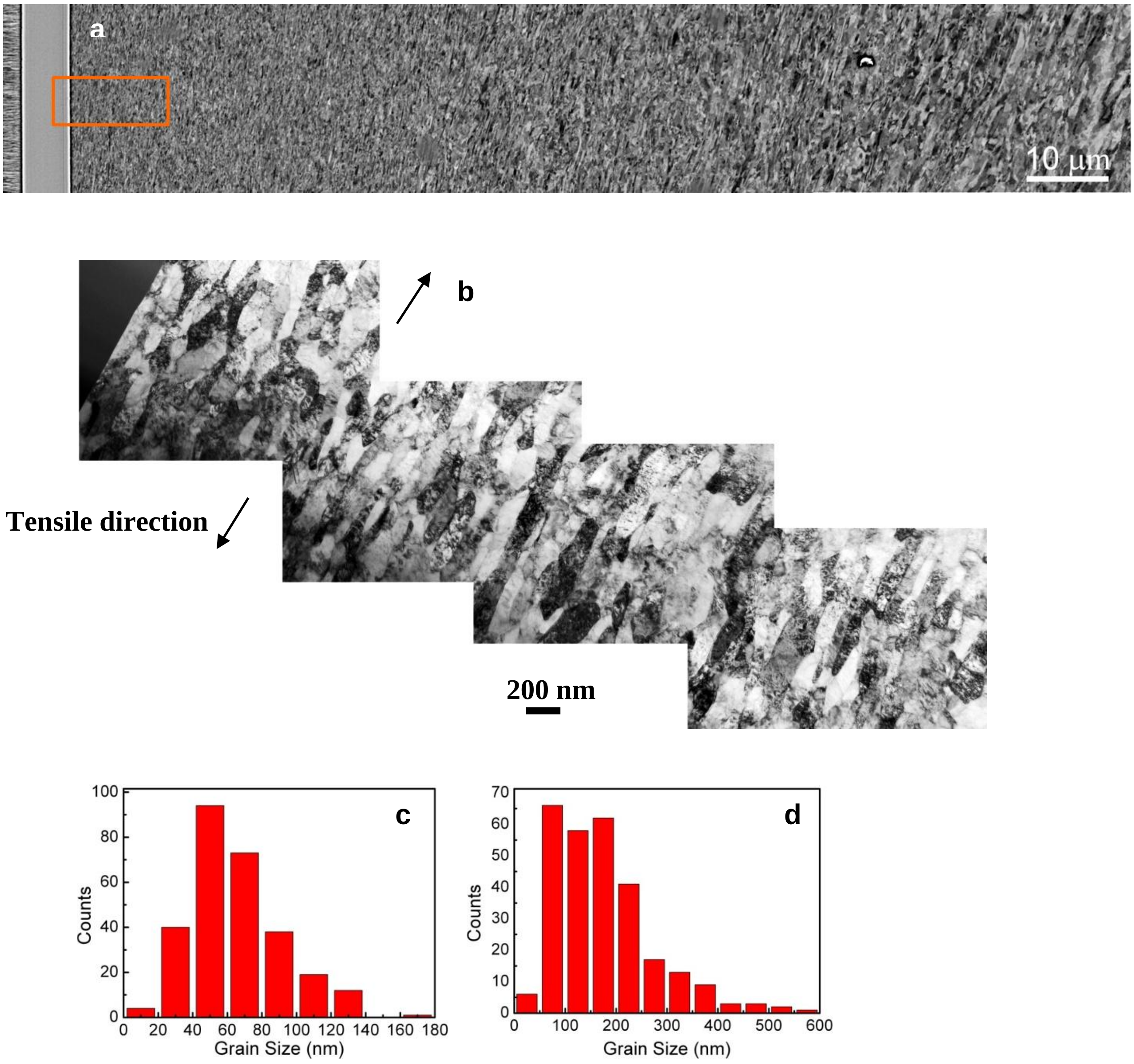

Figure S4. A cross-sectional structure of GNG structures before tension test. SEM image (a) and TEM image (b) of the GNG structure just beneath the Ni-P amorphous film. An elongated grain morphology, which is parallel to the tensile loading direction, was clearly seen after SMGT process. The grain size distributions from TEM measurements are statistically shown from transverse direction in (c) and from longitudinal direction in (d). The longitudinal axe is defined as the longest chord in one grain, which mostly parallel to tensile direction in (b). And the transverse axe is the vertical direction of the longitudinal direction.

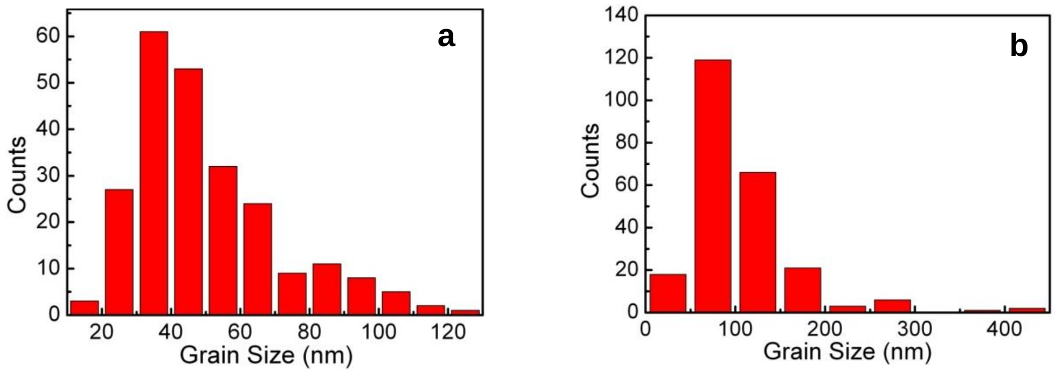

Figure S5. The grain size distributions of the GNG layer from TEM measurements after tension test. (Figs. 4a&b) are statistically shown from transverse direction in (a) and from longitudinal direction in (b).The statistical data counted from 236 grains. The grain size distributions are roughly comparable for the GNG layers before and after 15% tensile strain, from both the transverse direction and longitudinal direction. The interface became rougher compared to that before tension (Fig S4.a), yet the film is still adhered firmly to the substrate and no delamination was observed.

References:

1 Li, W. L., Tao, N. R. & Lu, K. Fabrication of a gradient nano-micro-structured surface layer on bulk copper by means of a surface mechanical grinding treatment. Scr. Mater. 59, 546-549, (2008).

2 Goldenstein, A. W., Rostoker, W., Schossberger, F. & Gutzeit, G. Structure of chemically deposited nickel. J. Electrochem. Soc. 104, 104-110, (1957).

3 Allen, R. M. & VanderSande, J. B. The structure of electroless ni---p films as a function of composition. Scr. Metall. 16, 1161-1164, (1982).

4 Keyse, R. J. & Hammond, C. Structure and morphology of electroless ni-p deposits. Mater. Sci. Technol.-lond. 3, 963-972, (1987).
